# Practical Notes and Formulæ

**Published:** 1877-11-20

**Authors:** 


### Practical Notes and Formulæ.

HYDROBROMIC ACID IN PRESCRIPTIONS.—Dr. D. C. Wade. (*Detroit Med. Jour.*) suggests the following method for preparing hydrobromic acid, and the several formulæ annexed:

DILUTE HYDROBROMIC ACID.

I. Bromide of potassium........120 grains.
Chrystalized tartaric acid....153 grains.
Water.......... .. .....1 fluid ounce.

Dissolve the salt and then the acid in water, and place in cold water for several hours, or until precipitation ceases, and decant. The results of the reaction are the formation of bitartrate of potassiam (cream of tartar), which is nearly insoluble, and sufficiently pure hydrobromic acid diluted with water, each fluid drachm of which contains ten grains of bromine. By preserving this proportion,any quantity can just as readily be made.

The affinity of hydrobromic acid for bases is between that of hydrochloric and hydriotic acids. I have prescribed it most frequently in half drachm doses well diluted.

III. Dilute hydrobromic acid.
Syrup..........of each one fluid ounce.

Mix and write—Teaspoonful in water,

This is not unpleasant to the taste, and may be given to obtain the constitutional effects of bromine as usually administered in combination with a base. It also acts like other mineral acids in being tonic, refrigerent, solvent, alterative, etc., and is very useful in the "bilious" conditions, including fevers, where the morbid symptoms recede with the coating on the tongue. I use little else in remittent fever.

IV. Sulphate of quinia....15 to 80 grains.
Dilute hydrobromic acid.
Syrup........of each one fluid ounce,

Mix and write—Teaspoonful in water.

This is extremely bitter, and in this respect cannot be improved by other additions. Like other acidulous preparations it is incompatible with licorice. Bromine has the power of modifying, in a marked degree, the cerebral effects of quinine; hence the value of this combination, aside from the alterative and other properties of the acid. In all cases of intermittent fever, I continue an antiperiodic from ten to thirteen days after the paroxysm ceases, and for permanent and other satisfactory results, this combination has proved to be far superior in my hands to any other not contained in the acid.

V. Sulphate of cinchonia....15 to 45 grains.
Dilute hydrobromic acid.
Syrup..........of each one fluid ounce.

Mix and write—Teaspoonful in water.

I can discover no difference in the effects of cinchonia and quinine, except that the latter is to be preferred as a stimulant. I prescribe cinchonia because of its cheapness.

VI. Red iodide of mercury........1 grain.
Dilute hydrobromic acid....1 fluid oz.
Fluid extract of orange peel.
Syrup......of each four fluid drachms

Mix and write—Teaspoonful in water.

The iodide of mercury is decomposed, the bromide being formed with the elimination of the iodide in the form of hydryotic acid. Mercury may be given in this manner for a long time without producing ptyalism, the salt being rapidly excreted.

VII. Fluid extract of ergot.
Syrup....of each four fluid drachms.
Dilute hydrobromic acid....1 fluid oz.

Mix and write—Teaspoonful in water.

HYDROBROMIC ACID IN CEREBRAL HYPERÆMIA.—I do not believe that any other combination equals this for efficiency in cases of cerebral hyperæmia. It is not only indicated where venesection would appear beneficial, but it may be administered by enema in a case of intercranial hæmorrhage, with the likelihood of arresting the transfusion, by capillary restriction, when an additional depletion of the arterioles, by artificial abstraction of blood, would still further endanger life without influencing the hæmorrhage, and is consequently positively contraindicated.

HYDROBROMIC ACID IN VERTIGO AND EPILEPSY.—Ergot and hydrobromic acid will be found to be promptly useful in the vertigo of plethora, with confusion of ideas, or where a determination of blood to the brain is prone to occur from other causes.

VIII. Fluid extract of stramonium....160 drops.
Dilute hydrobromic acid.
Syrup........of each one fluid ounce

Mix and and write—one half teaspoonful in water, the dose to be increased until the specific effects of the stramonium are marked, and there to be maintained.

I offer this combination as a prescription for epilepsy. I will simply say of it, that its effects in this disease are remarkable, and I think I have reason to consider it superior to any other plan of medication.

FOR ACUTE BRONCHITIS.

IX. Tartar emetic............2 grains.
Denarcotized tincture of opium....2 fluid drachms.
Dilute hydrobromic acid....1 fluid ounce.
Syrup, to make.......2 fluid ounces.

Mix and write—Teaspoonful in water.

AS A TONIC AND NERVINE.

X. Syrup of bromide of iron..........4 fluid drachms.
Bromide of quinia .......16 grains.
Dilute hydrobromic acid .......1 fluid ounce.
Syrup................4 fluid drachms.

Mix and write—Teaspoonful in water.

The wide applicability of this tonic is readily suggested by its composition.

Hydrobromic Acid in Nervous Dyspepsia.—

XI. Sub-carbonate of bismuth,..80 grains.
Dilute hydrobromic acid,..1 fluid ounce.
Dissolve and add
Saccharated pepsine,......80 grains.
Syrup, to make............2 fluid ounces.

Mix, filter and write—Teaspoonful in water.

This is preferable to ammoniated citrate of bismuth with pepsine, because it is not only permanent in the bottle, but it is not precipitated in the stomach, as is the citrate. Its indications are evident to the professional reader. To it may be added pancreatine, with or without the pepsine.

I trust I have shown by the foregoing formulas how readily bromine may be exhibited in an elegant manner, combined with other well known remedies. These formulas, however, are only intended as skeletons, upon which a great variety of changes may be rung to suit the "notions" of the prescibper.

Elixirs instead of syrups may be substituted, and additions of flavors may be made to render the medicine mor palatable. The doses given are for adults, and the frequency of their repetition in each case is to be determined in accordance with the circumstances.

Sedation is an indication in almost all diseased conditions, not only as a palliative, but to finally obtain radical results, by the greater curative efforts nature may make in the absence of irritation, aided, if necessary, by artificial means. It is no surprise, then, that the only known mineral sedative, being also a powerful alterative, should have found so prominent a place in the medical art; yet taking into consideration the facts that the salts of this drug are the preparations of it almost universally prescribed, and that they must be decomposed in the stomach, and hydrobromic acid produced before the effects of bromine can be obtained in any case, and that these salts cannot conveniently be combined with other medicines, it is an easy matter to gain the impression, that in the light of our present knowledge, there are too many who follow a very clumsy and unscientific method of exhibiting one of our most valuable therapeutic agents.—*Detroit Medical Journal.*

---

Scraps of Practice.—We are indebted to Dr. J. R. Bristow, Farmington, Texas, for the following notes or "*Clinical Scraps.*"—Case 1. Mrs. F., aged thirty-five, nursing fifth child about ten months old. Attention called to a tumor in the breast size of a partridge egg. Tumor isolated and well defined, painless, no interference with function of gland. Pronounced it innocent enlargement of one of lacteal glands, and advised stimulant liniment, with friction to the part. After giving directions in regard to tumor she said that she had had a like tumor in the same breast when nursing a former child, and it disappeared when the child was weaned. Are these tumors frequent? I do not now recall an allusion to them in medical literature.—B.

*Peculiar Insanity.*—Case 2. Mr. J., aged twenty-three. Was called to see him in third week of typhoid fever. Case progressed well for four days, and patient seemed convalescent; fever had ceased entirely, and appetite was returning. While sitting in the sick room one Sabbath evening with every indication of returning health, we were surprised at the exuberant spirits of patient. His mother said she never saw him more lively; but in the whole affair there was an incongruity that led me to suspicion the integrity of his intellect, apart from any delirium incident to the disease. On inquiry, found that his father had died in the asylum, and several of his near relatives had suffered from acute attacks of insanity. These facts gave me the key to much that he said and did in the next ten days, when he was sufficiently recovered to be moved, and passed from under my observation. Reflection: I do not remember to have seen, before or since, insanity complicated with the delirium of typhoid fever. Who has? Hydrate chloral and bromide potassium in equal quantities answered a better purpose in securing rest than opium, belladonna, or henbane.

---

*How to give Podophyllin.*—In purgative doses podophyllin is a dangerous drug. It is not only a powerful cathartic, irritant to the bowels, but it also has a powerful effect on the reflex nerves of the stomach, producing a most sickening nausea, and sometimes painful vomiting. I question whether any good accomplished by it as a cathartic compensates for the sickness and prostration it produces. But in very small doses it supplies a place not so will filled by any drug. In ⅛ to ¼ grs. doses it may be used even with infants, and is a powerful corrector of the acid, yeasty stools we so often meet in summer and autumn. As a substitute for mercury in adults, where the occupation compels activity and exposure, it is invaluable. In such cases ¼ gr. with rhubarb or aloes, or both, every three hours until a free catharsis, answers all the needs of mercury without its dangers. B.

